# Responses of zooplankton body size and community trophic structure to temperature change in a subtropical reservoir

**DOI:** 10.1002/ece3.5718

**Published:** 2019-10-29

**Authors:** Xiaofei Gao, Huihuang Chen, Lynn Govaert, Wenping Wang, Jun Yang

**Affiliations:** ^1^ Aquatic EcoHealth Group Key Laboratory of Urban Environment and Health Institute of Urban Environment Chinese Academy of Sciences Xiamen China; ^2^ Fujian Key Laboratory of Watershed Ecology Institute of Urban Environment Chinese Academy of Sciences Xiamen China; ^3^ University of Chinese Academy of Sciences Beijing China; ^4^ Department of Evolutionary Biology and Environmental Studies University of Zurich Zürich Switzerland; ^5^ Department of Aquatic Ecology Eawag: Swiss Federal Institute of Aquatic Science and Technology Dübendorf Switzerland; ^6^ Laboratory of Aquatic Ecology, Evolution and Conservation KU Leuven Leuven Belgium

**Keywords:** community ecology, plankton, subtropical reservoir, temperature, trait variation, trophic structure

## Abstract

Understanding the effects of global warming on trait variation and trophic structure is a crucial challenge in the 21st century. However, there is a lack of general patterns that can be used to predict trait variation and community trophic structure under the ongoing environmental change. We investigated the responses of body size and community trophic structure of zooplankton to climate related factors (e.g., temperature). Isotopic niche breadth was applied to investigate the community trophic structure across a 1‐year study from a subtropical reservoir (Tingxi Reservoir) in southeastern China. Body size and community isotopic niche breadth of zooplankton were larger during water mixing than stratification periods and correlated significantly with water temperature change along the time series. The contributions of intra‐ and intertaxonomic components to body size and community trophic structure variation showed significant relationships with the temperature change going from the mixing to stratification periods. Water temperature imposed direct effect on body size, while direct and indirect effect on the community trophic structure of zooplankton occurred through trophic redundancy along time series. Water temperature and community properties (e.g., body size, trophic redundancy, or trophic interaction) showed complex interactions and integrated to influence community trophic structure of zooplankton. Our results can expand the knowledge of how elevated temperature will alter individual trait and community trophic structure under future climate change.

## Graphical abstract

The stable isotope analysis, community‐wide metrics, and eco‐evolutionary partitioning metrics revealed that warming influenced the intra‐taxonomic and inter‐taxonomic contributions to the zooplankton body‐size and niche‐space variations in a deep reservoir. Our results highlight the importance of time‐series study and promote the understanding of responses of plankton ecosystem processes to global warming.

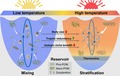

## 1 INTRODUCTION

In an increasingly human‐modified world, there is a growing urgency to understand how the trophic structure and biotic interactions are influenced by global warming (Dézerald et al., 2018; Rosenblatt & Schmitz, 2016). By the mid‐21st century, global surface temperature is predicted to increase by 2°C (Pachauri et al., 2014). As a consequence, this may alter local species interactions and the realized ecological niche of species (Pachauri et al., 2014). Niches represent the integration of all the interactions (e.g., trophic) with other species (Newsome, Martinez del Rio, Bearhop, & Phillips, 2007). They have been used to enhance our understanding of biotic interactions and many aspects of community structure (Dézerald et al., 2018; Layman et al., 2012; Newsome et al., 2007).

However, ecological niches are not easy to quantify because there exist many concepts of niche (Leibold, 1995) and these often have an abstract definition (Hutchinson, 1957; Newsome et al., 2007). One way of quantifying ecological niche is by using stable isotope analysis. Stable isotope analysis has been increasingly applied to investigate structure and functioning of animals (Catry et al., 2016) or to assess intraspecific and interspecific variation (Lemmens et al., 2017) by providing quantitative information on both resource and habitat uses (Fry, 2006). This information is commonly utilized to define ecological niche space (Layman, Arrington, Montaña, & Post, 2007; Newsome et al., 2007). Carbon and nitrogen stable isotopes (δ^13^C and δ^15^N) are most commonly used in studying food‐web structure (Fry, 2006; Layman et al., 2012; Post, 2002). The multivariate space given by δ^13^C and δ^15^N can be termed “isotopic niche” (Newsome et al., 2007), which is a low‐dimensional specification of Hutchinson's niche (Hutchinson, 1957; Yeakel, Bhat, Elliott Smith, & Newsome, 2016). The isotopic niche is increasingly used as a powerful proxy for assessing ecological niche in freshwater ecosystems (Dézerald et al., 2018; Lemmens et al., 2017).

Both intraspecific and interspecific variation can alter community trophic structure and dynamics (Bolnick et al., 2011; Dézerald et al., 2018; Griffiths, Petchey, Pennekamp, & Childs, 2018; Newsome et al., 2007). For example, a study by Lemmens et al. (2017) found that the differences in community isotopic niche space of fish were largely determined by intraspecific variation. In another example, Griffiths et al. (2018) found that a strong coupling between resources, ecological predator‐prey interactions, and intraspecific trait (i.e., body size) variation influenced community stability in experimental microcosms of protist communities. This indicates that a complex interplay exists between intra‐ and interspecific processes in temporal community dynamics. Similarly, abiotic environmental conditions (such as elevated temperature) may alter intra‐ and interspecific variation, thereby resulting in different community trophic structure and functioning of aquatic ecosystems (Rosenblatt & Schmitz, 2016; Sheridan & Bickford, 2011).

Studies looking at the effects of warming, researchers often use body size as a key trait. This is because body size is a crucial trait driving interspecific relationships, thereby determining the trophic structure and dynamics of communities (Elton, 1927; Merckx et al., 2018). Here, we focus on body size of zooplankton. Zooplankton play a significant role in controlling the physical and biogeochemical structure of aquatic ecosystems (Brierley, 2014; Hutchinson, 1961) and provide a critical link between phytoplankton through top‐down control and higher trophic levels via energy transfer (Carpenter, Kitchell, & Hodgson, 1985; Hannides, Popp, Landry, & Graham, 2009; Lin et al., 2017). Thus, understanding how temperature change will alter body size and community trophic structure of zooplankton is essential for gaining insight into how climate change will alter aquatic ecosystems and food webs. Normally, warming can lead to elevated metabolic rates and energy costs to sustain a given body size and is expected to favor smaller organisms (Sheridan & Bickford, 2011; Yvon‐Durocher, Montoya, Trimmer, & Woodward, 2011). However, a recent study has found that the general trend toward smaller organisms may sometimes be overruled by contrasting shifts in body size that critically depend on the association between body size and dispersal for different animal taxonomic groups (Merckx et al., 2018). In fact, the latter study only used intertaxonomic trait values, ignoring potential intrataxonomic effects.

Understanding the contribution of intraspecific and interspecific variation on trophic structure of consumers is important but still largely unknown (Griffiths et al., 2018; Laughlin & Messier, 2015; Lin et al., 2017). Community difference can be partitioned into species sorting (SS), intraspecific trait variation (ITV), and species turnover (ST) using the Price equation (Price, 1970). This method can help identify the relative importance of intra‐ and interspecific variation on community trophic structure and body size between ecosystems with contrasting ecology (Govaert, Pantel, & De Meester, 2016; Lemmens et al., 2017). However, how these processes interact at the low‐taxonomic level is less well‐studied. In this study, we use zooplankton data obtained from a subtropical reservoir (i.e., Tingxi Reservoir) in China to investigate the effects of biotic interactions, abiotic environment, body size, and their interactions on community trophic structure of zooplankton.

Water stratification is typically characterized by a clear boundary separating the oxic and anoxic zones under warm season, but the cooling surface water during cold period forces the water mixing and homogenize the water properties (Yu, Yang, Amalfitano, Yu, & Liu, 2014). The mixing and stratification periods are characterized by among others a difference in temperature in our study (Table 1). Therefore, the temporal analyses from the mixing to stratification periods were suitable for investigating the effects of temperature on the trait variation and community trophic structure in deep reservoirs. Specifically, we investigated the trophic structure and body size of zooplankton in a subtropical reservoir during water mixing and stratification periods using stable isotope analyses based on a 20‐day sampling frequency. Our key aim was to assess the key drivers contributing to the observed change in the community trophic structure and body size of zooplankton along this “natural” environmental gradient, translating into the following three questions:

1. Which consequences do the altered environmental conditions, such as elevated temperature, from water mixing to stratification periods have for body size and trophic structure in zooplankton communities?
2. If a change in zooplankton community body size and trophic structure is detected, is this due to inter‐ or intra‐taxonomic variation, and can it be related to the observed change in temperature?
3. How do direct and indirect effects of water temperature, other environmental change, and body size integrate to influence community trophic structure of zooplankton?

**Table 1:** PERMANOVA analysis characterizing environmental differences between the mixing and stratification periods in Tingxi Reservoir

Environmental conditions	Mixing versus stratification	
	*R* ^2^	*p*
Water temperature (°C)	0.716	**0.005**
Precipitation (mm)	0.003	0.544
pH	0.053	0.069
Dissolved oxygen (mg/L)	0.079	**0.044**
CO_2aq_ (μmol/L)	0.001	0.733
Chlorophyll *a* (μg/L)	0.004	0.502
Electrical conductivity (μS/cm)	0.032	0.111
Oxidation‐reduction potential (mV)	0.007	0.374
Euphotic depth (m)	0.014	0.250
Total carbon (mg/L)	0.016	0.234
Dissolved inorganic carbon (mg/L)	0.025	0.156
Total nitrogen (mg/L)	0.001	0.713
Nitrate and nitrite nitrogen (mg/L)	0.001	0.910
Ammonium nitrogen (mg/L)	0.020	0.200
Total phosphorus (mg/L)	0.001	0.852
Phosphate phosphorus (mg/L)	0.003	0.559
Total nitrogen:total phosphorus	0.006	0.444
Residuals	0.019	

Note

Note that no significant effect of time (*R* ^2^ = 0.096, *p* = 0.075) on the overall environmental change. Significant *p*‐values are indicated in bold.

## 2 MATERIALS AND METHODS

### 2.1 Study area

The field sampling was conducted in the lacustrine zone close to the dam of Tingxi Reservoir (24°47′N, 118°08′E) near Xiamen city, Fujian province, southeast China. Xiamen is characterized by a subtropical humid monsoon climate, with an annual mean precipitation of 1,335.8 mm and an annual mean temperature of 20.7°C (Liu et al., 2015). Tingxi Reservoir is built on a tributary of Dong Xi River, the largest river of Xiamen city. The main functions of the reservoir are flood control, irrigation, and water supply. The reservoir has a well‐forested catchment and almost no aquatic macrophyte in the waters possibly caused by large fluctuations of water level. It normally experiences water mixing and stratification periods with a mean surface water temperature of 18.97 and 27.11°C, respectively (Figure S1).

### 2.2 Sample collection and processing

In this study, particulate organic matters (POM) were collected from surface and bottom waters every 20 days from November 2015 to December 2016. POMs were partitioned into three fractions: pico‐POM (0.2–3 μm), nano‐POM (3–20 μm), and micro‐POM (20–200 μm). At least three replicates were collected for each sample.

Water samples were collected from surface (0.5 m below surface water) and bottom layers (hypoxic boundary or 2 m above the sediment; Figure S1). A total of 100 L were prefiltered in situ through 200 and 20 μm nylon sieves. An additional 30 L of water was immediately taken back to the laboratory and filtered sequentially through 20, 3, and 0.2 μm pore‐size polycarbonate filters (47 mm diameter; Millipore). The samples were then gently backwashed from filters into 15 ml plastic centrifuge tubes. All the samples were then freeze‐dried at −80°C prior to nitrogen and carbon elemental and isotopic analysis.

Zooplankton were sampled with a plankton net (mesh size 112 μm) along the water column by hauling vertically from the bottom (2 m above the sediments) to surface waters 20 times. After 12 hr starvation and gut evacuation treatment in deionized water, zooplankton individuals were filtered with 200 μm nylon sieve before gently backwashed into centrifuge tubes. In this study, zooplankton were classified into six dominant taxonomic groups based on taxonomy and size: *Bosmina*, *Bosminopsis*, other Cladocera, small Cyclopidae (200–450 μm), large Cyclopidae (≥450 μm), and Diaptomidae. All zooplankton taxonomic groups were identified and counted on a Nikon SMZ800 stereo microscope (Nikon Corporation). The main species included in each group are listed in Table S1. About 50 copepods and 200 cladocerans were picked up from each replicate to get enough biomass for the isotopic analysis. Those individuals were directly put into pressed tin capsules. Then, we got dry weight of individual zooplankton taxa (DW, μg/ind) after drying in an oven at 60°C for 48 hr. The relationship between ecological drivers and body size was investigated using the dry weight of zooplankton taxa following a previous study (Yvon‐Durocher et al., 2011). Finally, all samples were stored in a desiccator until carbon and nitrogen elemental and isotopic analysis.

### 2.3 Environmental data

Water temperature (Temp), pH, dissolved oxygen (DO), electrical conductivity (EC), and oxidation‐reduction potential (ORP) were measured in situ at 0.5 m interval using a multiparameter water quality analyzer (Hach Company). Chlorophyll *a* (Chl *a*) was measured with a PHYTO‐PAM Phytoplankton Analyzer (Heinz Walz GmbH). Total carbon (TC), dissolved inorganic carbon (DIC), total nitrogen (TN), nitrate and nitrite nitrogen (NO_x_‐N), ammonium nitrogen (NH_4_‐N), total phosphorus (TP), and phosphate phosphorus (PO_4_‐P) were analyzed following the standard methods (Greenberg, Clesceri, & Eaton, 1992). Dissolved CO_2_ (CO_2aq_) was calculated from a function of dissolved inorganic carbon (DIC) concentration, water temperature, and pH (Wetzel & Likens, 2000). Each of these environmental variables was averaged over the depth that was consistent with zooplankton samples over the entire sampling period. Precipitation data were taken from the China Meteorological Data Service Center (http://data.cma.cn) (Xiamen ID 59134; coordinates 24°29′N, 118°04′E). Water transparency was measured with a 30‐cm‐diameter Secchi disk. Euphotic depth (Zeu) was estimated as 2.7 times of the Secchi depth (Cole, 1994). We characterized the environmental differences between the two periods (the mixing vs. stratification periods) based on the PERMANOVA in R environment (R Core Team, 2017).

### 2.4 Stable isotope analysis

The POM samples were ground to a fine powder. In total, 1.2–1.5 mg pico‐ and nano‐POMs of each replicate were put into the pressed tin capsules, while 0.2–0.5 mg micro‐POM were also prepared. Percentage carbon, percentage nitrogen, and stable isotopic ratios were measured using the Thermo Electron Flash EA 2000 Elemental Analyzer (EA) coupled to a Delta V isotope ratio mass spectrometer (IRMS) (Thermo Fisher Scientific Inc.). We abstained from acidification because it has no significant effect on carbon isotope, but it can affect nitrogen determinations (Marcus, Virtue, Nichols, Meekan, & Pethybridge, 2017). Carbon and nitrogen stable isotope ratios were presented as δ^13^C and δ^15^N relative to Vienna Pee Dee Belemnite carbonate and atmospheric N_2_ isotope, respectively (Fry, 2006). Prior to statistical analyses, we used carbon content and C:N ratios to correct δ^13^C of zooplankton for lipid bias (Syväranta & Rautio, 2010).

### 2.5 Data analysis

#### 2.5.1 Body size and trophic structure variations of zooplankton from the mixing to stratification periods

To answer our first research question to determine variation in zooplankton body size and trophic structure from the mixing to stratification periods, we evaluated changes in body size (dry weight, DW), range of niche diversification (δ^13^C), and vertical structure of the food web (δ^15^N) along the time series. We also assessed the contributions of potential food sources to zooplankton community and compared the trophic structure of zooplankton between the mixing and stratification periods. We further explored the different responses of body size and trophic structure to environmental factors between the mixing and stratification periods.

##### Contributions of potential food sources to zooplankton during the mixing and stratification periods

In general, zooplankton eat food resources smaller than themselves (Cohen, Jonsson, & Carpenter, 2003). Therefore, the pico‐, nano‐, and micro‐POMs were potential food sources for zooplankton. Bayesian stable isotope mixing models were applied to estimate the relative contributions of different food particles to zooplankton communities during water mixing and stratification periods, respectively. We applied an a priori Monte Carlo simulation of mixing polygons following Smith, Mazumder, Suthers, and Taylor (2013). Twelve and six zooplankton samples from water mixing and stratification periods, respectively, were removed based on the 95% confidence region of the simulated mixing polygon (Figure S2). The Bayesian mixing models were run for 20,000 iterations, 2,000 burn‐in, and a thinning interval of 20 based on the result of convergence diagnostics (Parnell & Inger, 2016). The first 2,000 iterations were discarded, and subsequent iterations were stored and used for the posterior distribution (Figure S3). We performed the mixing models using the *simmr* package (version 0.3) (Parnell & Inger, 2016) running in R (version 3.5.1) environment (R Core Team, 2017). Trophic fractionation factors of 0.4‰ (*SD* = 1.3‰) for δ^13^C and 3.4‰ (*SD* = 1.0‰) for δ^15^N were used, respectively (Post, 2002).

##### Differences in body size and community trophic structure between the mixing and stratification periods

We tested for differences in community‐weighted mean body size and taxonomic mean body size between the mixing and stratification periods using nonparametric Mann–Whitney *U* test. To assess differences in community trophic structure of zooplankton, we utilized community isotopic niche breadth, such as Layman metrics derived from δ^13^C and δ^15^N values (Layman et al., 2007). Because of the different sample size, we estimated the community metrics using recently developed Bayesian approaches (Jackson, Inger, Parnell, & Bearhop, 2011) implemented in the *SIBER* package (version 2.1.3) in R environment (R Core Team, 2017). The range of δ^13^C and δ^15^N provided information of basal resources diversification (CR) and trophic length (NR), respectively. The degree of trophic diversity was measured as the mean Euclidean distance of each sample to the δ^13^C‐δ^15^N biplots centroid (CD). The overall density of taxa packing and its evenness were measured by the mean nearest neighbor distance (MNND) and its standard deviation (SDNND), respectively (Layman et al., 2007). Lower values of MNND and SDNND represent higher trophic redundancy and more uniform distribution of trophic niches, respectively. The main index of isotopic niche breadth (Jackson et al., 2011) was analyzed by the standard ellipse area (SEA), which is the Bayesian equivalent of the total area of the δ^13^C‐δ^15^N biplot space (Layman et al., 2007). The variation in isotopic niche breadth of zooplankton was examined by the Bayesian standard ellipse areas (SEA_B_). The small sample size‐corrected standard ellipse areas (SEA_C_) were also calculated.

Many studies have shown that variation in isotopic values of basal resources can influence the consumer signals; thus, a baseline correction is necessary for the isotopic differences of resources between ecosystems when using Euclidean‐based metrics (Catry et al., 2016; Olsson, Stenroth, Nyström, & Graneli, 2009). We standardized zooplankton isotopic values by subtracting the specific mean values of food sources (contributing more than 12% during at least one of periods) and dividing by specific range value of the same sources.

##### Different responses of body size and trophic structure to environmental factors between the mixing and stratification periods

Before statistical analysis, all data were log‐transformed except pH and C:N ratio to assure homogeneity and normality of variables. We performed a redundancy analysis (RDA) to find the significant environmental factors accounting for the changes in body size (DW), δ^13^C and δ^15^N of zooplankton using CANOCO for Windows 4.5 for the mixing and stratification periods, separately. Before RDA, we only retained one of the environmental variables which have strong collinearity with others based on the Spearman correlations (*R* > 0.7, *p* < 0.05). Then the remaining environmental variables with variance inflation factors (VIF) < 10 were selected, and the significance of the conditional effects was evaluated with 999 Monte Carlo permutations. Based on the RDA, we assessed correlations between important explanatory environmental variable and body size (DW) of individual taxonomic groups, density of taxa packing (MNND), and isotopic niche breadth (SEA_B_) of zooplankton using linear regression analysis.

#### 2.5.2 Contributions of intra‐ and intertaxonomic group variations to zooplankton body size and community trophic structure

To answer our second research question to determine contributions of intra‐ and intertaxonomic group variations to community trophic structure, we used the Price equation as described in Govaert et al. (2016) and Lemmens et al. (2017); that is, $\Delta z\;=\;\sum z_{i}^{\prime }\;\left(q_{i}^{\prime }-q_{i}\right)\;+\;\sum q_{i}^{\prime }\;\left(z_{i}^{\prime }-z_{i}\right)\;+\;\sum q_{i}^{\prime }z_{i}^{\prime }-\sum q_{i}z_{i}$, where *z_i_* is the trait value for species *i* and *q_i_* is the relative abundance for species *i*. We only have measurements on the genus or family level; therefore, the contributions calculated reflect changes at the taxonomic level. Thus in this study, *z_i_* represents the trait value for taxa *i* and *q_i_* is the relative abundance for taxa *i*. The first two sums are taken across the shared taxa between two time points, while the last two sums are across the nonshared species of the second and first time point, respectively. The observed changes in SEA_B_, body size (DW) and density of taxa packing (MNND) of the zooplankton community from the mixing to stratification periods can then be partitioned into taxa sorting (TS), intrataxonomic group variation (ITV), and taxa turnover (TT) as opposed to species sorting, intraspecific trait variation, and species turnover as described in Lemmens et al. (2017). In this partitioning method, TS reflects a community change due to a shift in relative abundances of the different taxonomic groups and may be mediated by environmental factors. We also explicitly took into account TT, taxa gain and loss, which represent that part of community change due to a different set of taxonomic groups present between two sampling points. TT here reflects both relative abundances and biomasses of zooplankton taxa lower than 3%. Last, ITV represents that part of the community change due to within taxa changes which can encompass shifts in species abundances, evolutionary change, environmental responses (e.g., phenotypic plasticity), or ontogenetic changes (Govaert et al., 2016; Violle et al., 2012). The partitioning approach was applied to a change in the body size, MNND, and SEA_B_ of zooplankton community from the mixing to stratification periods to get the contribution of each component to the overall mean change between the two periods. We also performed a partitioning to the observed temporal differences in body size, δ^13^C, and δ^15^N of the zooplankton along the sampling points. The mean relative abundance, body size, and MNND values of each zooplankton taxonomic group were used in the partitioning analysis. To determine whether these contributions varied with environmental change, we used the Spearman correlations between the relative contributions of these components and the change in different environmental variables.

#### 2.5.3 The direct and indirect effects of ecological drivers on zooplankton body size and community trophic structure

To answer our third research question, the direct and indirect effects of biotic and abiotic factors on the body size and SEA_B_ of zooplankton community were analyzed using a partial least squares path modeling (PLS‐PM) in the package *plspm* (version 0.4.9) in R (R Core Team, 2017). The variables used in the PLS‐PM analyses were the significant factors obtained from the RDA for the mixing and stratification periods described earlier. These significant environmental variables were divided into four block variables: water temperature, physicochemical factors (Zeu, EC, CO_2aq_ and DO), nutrients (TN, NO_x_‐N, TP, and DIC), and chlorophyll *a*. Here, we did not consider the complex interactions between temperature, nutrients, and physicochemical variables. We just explored how these block variables were related to body size (DW), MNND, and SEA_B_ of the zooplankton community. Body size of *Bosminopsis* and small Cyclopidae in the mixing period were removed, because they had strong collinear relationship with large Cyclopidae (Spearman correlations; *r* = 0.77, *p* < 0.05) and *Bosmina* (*r* = 0.75, *p* < 0.05), respectively. Similarly, body size of *Bosminopsis* and large Cyclopidae in the stratification period were removed from the body size block variable. We performed an initial PLS‐PM structure equation to remove the variables with loadings < 0.7 and then performed the final PLS‐PM structure equation with the remaining variables (Wang, Pan, Soininen, Heino, & Shen, 2016). We quantified the relationships among these block variables with path coefficients. The goodness of fit index (GoF) and *R* ^2^ were used to estimate the prediction performance of models.

## 3 RESULTS

### 3.1 Body size and trophic structure variations of zooplankton from the mixing to stratification periods

We found pronounced seasonal variation in δ^13^C, δ^15^N, and body size of zooplankton taxonomic groups (Figure S4). The δ^13^C varied similarly in range between the mixing and stratification periods; however, it strongly increased during the mixing period, while a gradual decrease was found during the stratification period. The δ^15^N, however, showed larger variation in the mixing period (ranging from 2.9 to 18.9‰) compared with the stratification period (ranging from 0.7 to 10.4‰).

**Figure 1:**
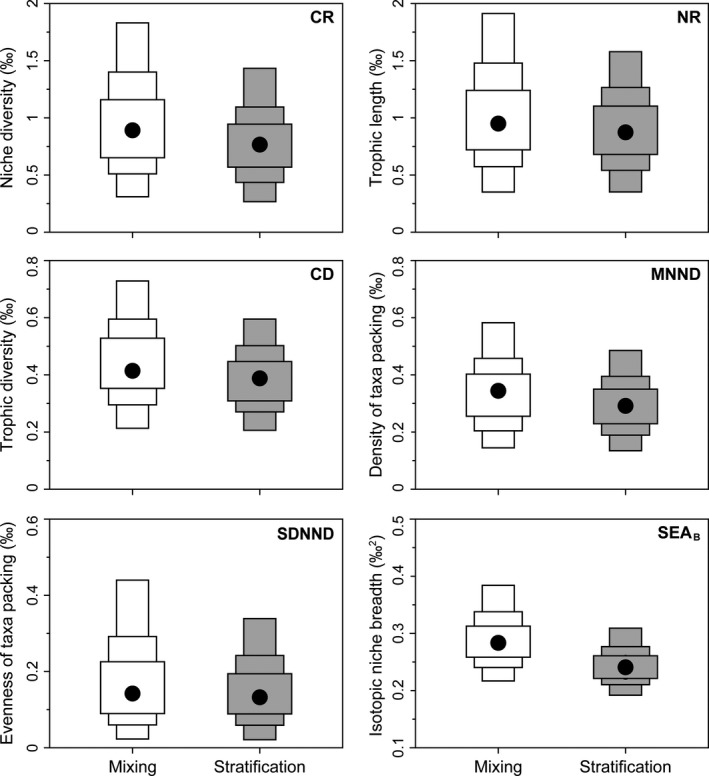
Bayesian Layman's community‐wide metrics and isotopic niche breadth (SEA_B_) for zooplankton community from water mixing (white box) and stratification (gray box) periods, respectively. Black dots represent the mode, and boxes represent the 50%, 75%, and 95% credibility intervals. CR, δ^13^C range reflecting niche diversification at the base of the food web; NR, δ^15^N range reflecting vertical structure of the food web; CD, mean distance to centroid reflecting an average degree of trophic diversity; MNND, mean nearest neighbor distance reflecting overall density of taxa packing; SDNND, standard deviation of nearest neighbor distance reflecting evenness of taxa packing; and SEA_B_, standard Bayesian ellipse area reflecting isotopic niche breadth

The mixing and stratification periods exhibited significant difference in water temperature at *p* < 0.01 and dissolved oxygen at *p* < 0.05 (Table 1), but also in the main food sources for zooplankton in surface water (Figure S5). Pico‐ and nano‐POMs were the main food sources of zooplankton community (10%–17%) in the mixing period, but surface pico‐POM became the main food source (73%) in the stratification period (Figure S5). Overall, the community average body size showed a significant decrease from the mixing to stratification periods (mean ± *SD*: 1.59 ± 0.20 μg/ind in the mixing period and 1.36 ± 0.45 μg/ind in the stratification period, respectively; nonparametric Mann–Whitney *U* test, *p* < 0.01). All, except two (small Cyclopidae and Diaptomidae), zooplankton taxonomic groups showed a significant decrease in body size (DW) going from the mixing to stratification periods. Small Cyclopidae showed a significant increase in body size, while no difference in body size was found for Diaptomidae (Figure S6). There was no strong difference for the niche diversity (CR), trophic length (NR), trophic diversity (CD), density, and evenness of taxa packing (MNND and SDNND, respectively) between the mixing and stratification periods (Figure 1). However, the community isotopic niche breadth (SEA_B_) was larger during the mixing period than stratification period (Figure 1). This was found for each taxonomic group individually, in which all zooplankton taxonomic groups showed a broader isotopic niche breadth in the mixing period than the stratification period (Figures S6–S7). Moreover, we also found a lower niche overlap between sampling time points in the mixing period than the stratification period (Figure S7). Similarly, all zooplankton taxonomic groups had a lower trophic redundancy (higher MNND values) in the mixing than stratification periods with an exception of *Bosminopsis* (Figure S8). The temporal isotopic niche breadth (SEA_B_) showed a strong correlation with the MNND during the mixing and stratification periods (*r* = 0.82 and *p* < 0.01 for mixing; *r* = 0.88 and *p* < 0.01 for stratification; Figure S9).

The altered environmental conditions between the mixing and stratification periods also resulted in different response patterns of body size and trophic structure to environmental factors (Figure S10). For example, during the mixing period, the body size was significantly correlated with chlorophyll *a* and dissolved CO_2aq_. However, temperature exhibited a significant negative effect on zooplankton body size and δ^15^N and a significant positive effect on zooplankton δ^13^C in the stratification period (Figure S10). Similarly, water temperature was significantly correlated with body size and δ^15^N of zooplankton along the time series (Figure S10).

**Figure 2:**
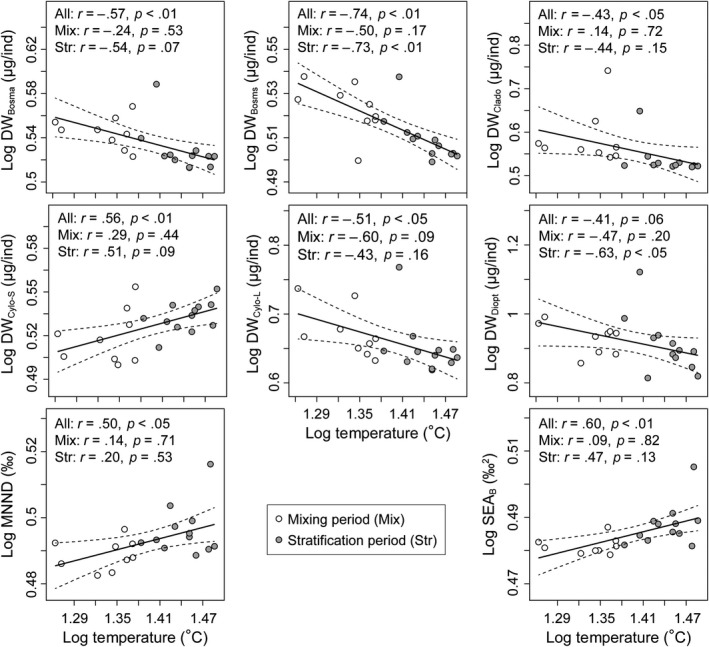
Significant relationships between water temperature and body size (DW) or isotopic niche breadth (SEA_B_) of zooplankton (*n* = 21) from the mixing to stratification periods. The continuous line represents a linear logistic regression curve with its 95% confidence interval (dashed line). All data were log‐transformed. DW_Bosma_, DW_Bosms_, DW_Clado_, DW_Cyclo‐S_, DW_Cyclo‐L,_ and DW_Diapt_ represent dry weight of *Bosmina*, *Bosminopsis*, other Cladocera, small Cyclopidae (200–450 μm), large Cyclopidae (≥450 μm) and Diaptomidae, respectively. The values for the isotopic niche breadth used for the mixing and stratification periods, respectively, represent the mode values of the Markov chain Monte Carlo (MCMC) output. MNND was the mean nearest neighbor distance calculated for each sample along the mixing and stratification periods

Therefore, we used linear regression to track the effect of water temperature on body size and isotopic niche breadth of zooplankton along the time series (Figure 2). Body size of *Bosmina*, *Bosminopsis*, other Cladocera, and large Cyclopidae decreased significantly with increasing water temperature (*r* = −0.57, *p* < 0.01; *r* = −0.74, *p* < 0.01; *r* = −0.43, *p* < 0.05; and *r* = −0.51, *p* < 0.05, respectively) from the mixing to stratification periods. However, small Cyclopidae showed an inverse trend and was significantly positively correlated with the water temperature (*r* = 0.56, *p* < 0.05). No significant effect of water temperature on body size of Diaptomidae was found (*r* = −0.41, *p* = 0.06) along the time series, although a significant and negative relationship was found in the stratification period (Figure 2). These results were in line with the nonparametric test for body size between the mixing and stratification periods (Figure S6). Our results also revealed a significant correlation between water temperature and density of taxa packing (MNND, *r* = 0.50, *p* < 0.05) and isotopic niche breadth (SEA_B_, *r* = 0.60, *p* < 0.01) of the zooplankton community, respectively, along the time series (Figure 2).

### 3.2 Contributions of intra‐ and intertaxonomic group variations to zooplankton body size and community trophic structure

**Figure 3:**
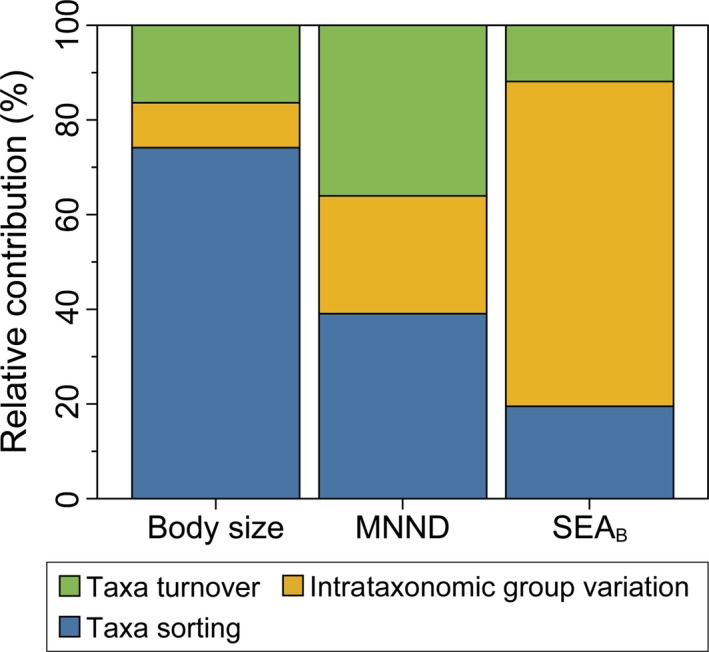
The relative contributions of taxa sorting (TS), intrataxonomic group trait variation (ITV) and taxa turnover (TT) to observed changes in body size (DW), the density of taxa packing (MNND), and isotopic niche breadth (SEA_B_) of zooplankton community going from water mixing to stratification periods, respectively

Our result showed that the intrataxonomic group trait variation (ITV) in niche space contributed 68.6% to the observed change in community isotopic niche breadth (SEA_B_) between water mixing and stratification periods. The contributions of taxa sorting (TS) and taxa turnover (TT) were 19.5% and 11.9%, respectively (Figure 3). However, TS was a main contributor to body size change of zooplankton (74.1%) between the mixing and stratification periods, indicating that there were stronger changes in taxonomic abundances than body size change within taxa between the two periods. For the change in density of taxa packing (MNND), TS and TT were the main contributors (39.1% and 36.0%, respectively; Figure 3). The partitioning for temporal changes in body size, δ^13^C, and δ^15^N between consecutive sampling points also illustrated the importance of TS to the change in body size, and ITV contributed more to changes in trophic structure of the zooplankton community (Figure S11).

We found that the intra‐ and intertaxonomic group variation contributions correlated with the environmental change (Figure S12). First, during the mixing period, both of the relative contributions of TS and ITV to δ^13^C were correlated significantly with the DO and NO_x_‐N variations between consecutive sampling points. The relative contribution of TS to δ^15^N was correlated significantly with CO_2aq_ variation, and TT to δ^13^C showed a significant relationship with chlorophyll *a* in the mixing period. Second, during the stratification period, the relative contributions of TT to body size (based on DW) and δ^13^C both showed a significant correlation with water temperature change between consecutive sampling points. We also found a significant correlation between nutrients (e.g., DIC and TP) variation and the relative contribution of ITV to body size in the stratification period. Third, for the time series from the mixing to stratification periods, the relative contributions of TS and ITV to body size were both correlated significantly with water temperature change. Similarly, the relative contributions of ITV to δ^13^C exhibited a significant correlation with changes in temperature and DO (Figure S12). The relative contributions of TS and ITV to δ^15^N were correlated significantly with the change in pH along time series (Figure S12).

### 3.3 The direct and indirect effects of ecological drivers on zooplankton body size and community trophic structure

**Figure 4:**
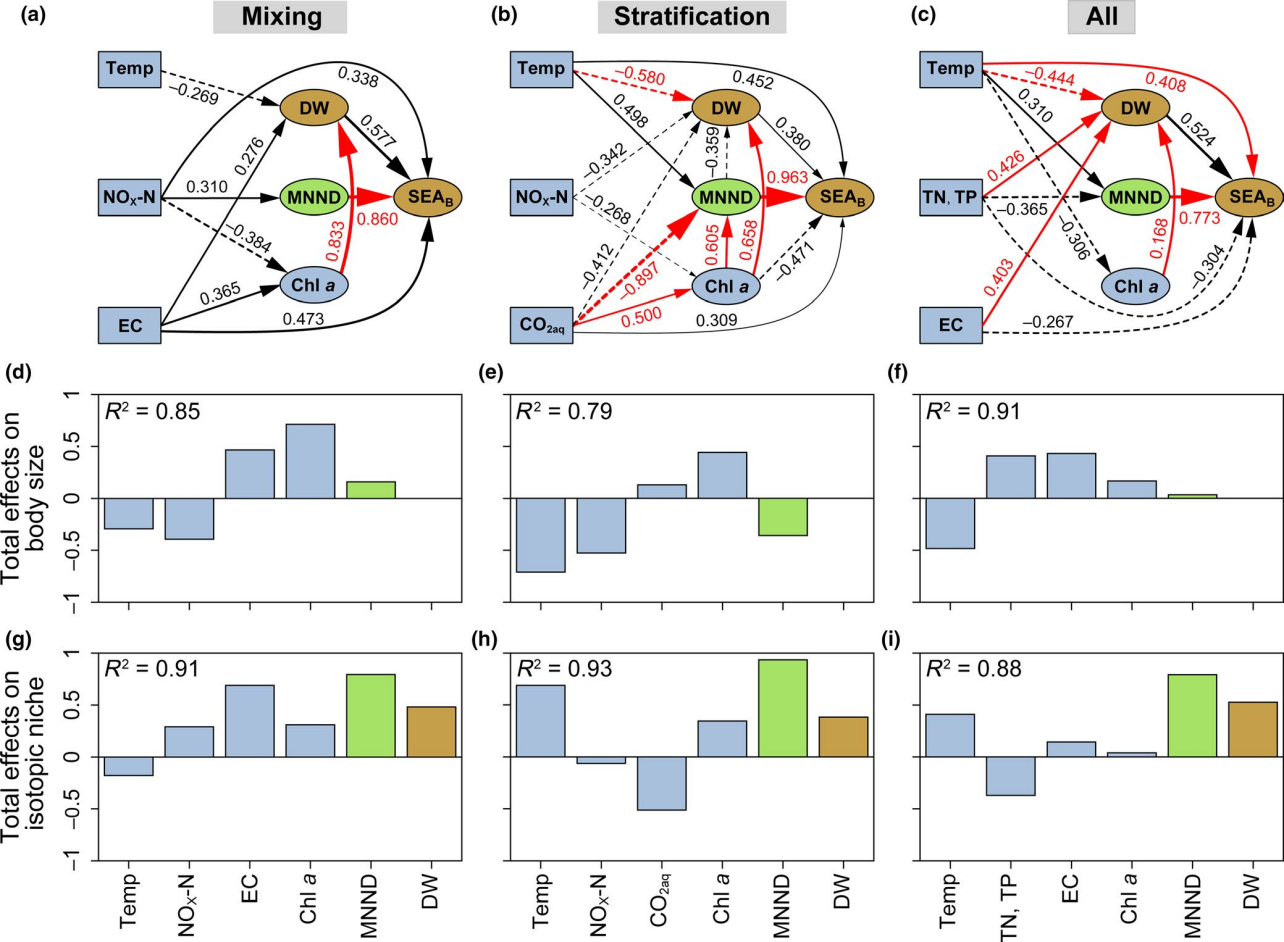
The final partial least squares path models (PLS‐PM) showing direct and total effects of significant environmental and biological (MNND) factors on body size (DW) and isotopic niche breadth (SEA_B_) of zooplankton community, analyzed separately for the mixing (a, d, g) and stratification (b, e, h) periods and for both periods (c, f, i). The environmental factors included water temperature (Temp), nutrient (NO_x_‐N for the mixing or stratification periods; TN and TP along all time series), physicochemical factors (EC and CO_2aq_ of the mixing and stratification periods, respectively; EC along time series) and chlorophyll *a* (Chl *a*). *R* ^2^ indicates the degree explained by their independent latent variables. Only paths with a coefficient > 0.25 are shown for simplicity. Solid and dashed lines represent positive and negative effects, respectively. Red lines represent significant paths (*p* < 0.10). The thickness of the line represents the absolute value of the path coefficients. The goodness of fit index for (a), (b), and (c) is 0.637, 0.663, and 0.607, respectively. All data were log‐transformed

We conducted PLS‐PM structure equation models to explore the direct and indirect effects of water temperature and other environmental changes on zooplankton body size and community trophic structure (here represented by SEA_B_). Body size (DW) and SEA_B_ were well explained by our block variables in the mixing (*R* ^2^ = 0.85 for body size and *R* ^2^ = 0.91 for community trophic structure) and stratification periods (*R* ^2^ = 0.79 and *R* ^2^ = 0.93, respectively), as well as along the whole time series (*R* ^2^ = 0.91 and *R* ^2^ = 0.88, respectively). The density of taxa packing (MNND) was the most important factor affecting the SEA_B_ of zooplankton community in both periods (Figure 4a,b,c). Chlorophyll *a* was a key factor significantly influencing body size of zooplankton (Figure 4a,b,c). During the mixing period, chlorophyll *a* was the main factor significantly influencing body size of zooplankton (Figure 4a,d). During the stratification period, water temperature became one of the strongest factors influencing body size of zooplankton (Figure 4b,e) and also exhibited an important contribution to SEA_B_ (Figure 4h). For the whole time series, water temperature exhibited a direct and significant effect on body size and SEA_B_ of zooplankton community (Figure 4c) and was the main contributor to the body size (Figure 4f). Overall, although body size was an important contributor to SEA_B_ in both periods (Figure 4g,h,f), the direct paths between them were nonsignificant (Figure 4a,b,c).

## 4 DISCUSSION

There is ample evidence that continued global warming will alter community trophic structure, and this may occur via intra‐ and interspecific processes (Sheridan & Bickford, 2011; Violle et al., 2012). In this study, we combined stable isotope analysis with a quantitative partitioning approach and a statistical path model to explore the complex relationship between water temperature, body size, and community trophic structure of zooplankton along time series in a subtropical reservoir. The time series data could be subdivided into two distinct periods: mixing and stratification periods, which were closely related with the temperature change. We found that water temperature change was the main contributor to the strong differences in environmental conditions between these two periods and that the altered environmental change consequently influenced body size and community trophic structure of zooplankton. Both community changes were, however, driven by different contributions of intra‐ and intertaxonomic components. Last, our path model demonstrated that both abiotic conditions and zooplankton properties shaped zooplankton trophic structure. Using time series data, we were able to explore the key ecological and environmental drivers on the trait and trophic structure variation of zooplankton and could offer some new insights into the links and interactions between warming, body size, and community trophic structure in a natural setting.

### 4.1 Body size and trophic structure variations of zooplankton from the mixing to stratification periods

We found shrinking body size with elevated temperature along time series from the mixing to stratification periods. The smaller zooplankton taxonomic groups found in the stratification period were in line with recent investigations that warming benefits smaller organisms (Chiba et al., 2015; Merckx et al., 2018; Yvon‐Durocher et al., 2011). We also found that taxonomic groups differed in their body size response to temperature, which might be due to different food adaptability and competitive ability (Hart & Bychek, 2011; Sheridan & Bickford, 2011). For example, temperature could influence body size and trophic structure through chlorophyll *a* and trophic interactions (e.g., trophic redundancy). Further, small zooplankton are typically competitively inferior than larger zooplankton under food‐limited conditions at higher temperature (Hart & Bychek, 2011). The heterogeneous size changes across different zooplankton taxa are likely to alter zooplankton community balances. However, the mechanisms and consequences of the observed size change are not yet fully understood (Sheridan & Bickford, 2011).

For the community trophic structure, we found smaller isotopic niche breadth of zooplankton with elevated temperature along time series (Figure 2). The wider isotopic niche breadth during the mixing period was due to large variation and almost no overlap in the isotopic niche distributions between the different sampling points. The isotopic niche breadth had no significant difference along the mixing period. Some studies have highlighted the importance of niche variation and trophic redundancy for the overall community stability across different ecosystems (Ojwang, Ojuok, Mbabazi, & Kaufman, 2010; Sanders, Thébault, Kehoe, & van Veen, 2018; Schwartz‐Narbonne et al., 2019). Therefore, the highly conserved niche with low level of trophic redundancy (high MNND values) in the mixing period can enhance ecosystem resilience from the stratification periods. But the exact mechanisms of niche variation are not well understood (Newsome et al., 2007; Schwartz‐Narbonne et al., 2019).

### 4.2 Contributions of intra‐ and intertaxonomic group variations to zooplankton body size and community trophic structure

The importance of intra‐ versus interspecific trait variation has been highlighted in aquatic ecosystems (Brans et al., 2017; Griffiths et al., 2018; Lemmens et al., 2017) and has been shown to play an important role in shaping the community trophic structure (Bolnick et al., 2011; Gibert & DeLong, 2017). Here, we assessed how intra‐ (ITV) versus intertaxonomic (TS) variations contributed to changes in community body size and trophic structure of zooplankton. Our results showed that ITV contributed substantially to the observed decrease in community isotopic niche breadth of the zooplankton from the mixing to the stratification periods, because each taxonomic group reduced its isotopic niche breadth with increasing water temperature. Although Lemmens et al. (2017) have shown the importance of intraspecific variation in fish community trophic structure, less is known for zooplankton. Because this was assessed at the taxonomic level, we cannot separate ITV‐induced changes due to species reducing their isotopic niche breadth, or due to shifts toward species with lower isotopic niche breadth. On the contrary, we found that the decrease in community body size was largely determined by taxa sorting (TS), indicating there was a shift toward smaller taxa in the stratification period. The low contribution of ITV to change in community body size can be explained by the varying response of different taxa to temperature change, which may result in a net effect of zero. This result also highlights that in order to understand community patterns to environmental change, it may be important to look at subcomponents (e.g., species or taxonomic groups) of the community.

Linking contributions of intra‐ and intertaxonomic group variation to observed environmental change might improve our understanding of zooplankton community mechanism when these processes may play an important role in determining community trophic structure. For instance, we found that the contribution of ITV to the changes in δ^13^C and body size significantly decreased with increasing water temperature along the time series (Figure S12). Zooplankton taxonomic groups with different body sizes typically have different abilities to regulate their physiological processes (e.g., metabolic rates and food size ranges; Hart & Bychek, 2011). Therefore, some zooplankton taxa might be too slow to change their physiology in response to the abrupt temperature change. While we have highlighted the importance of water temperature on body size and community trophic structure of zooplankton in the stratification period, we also found that temperature change potentially plays a key role on body size and community trophic structure through altering the ecological role of intrataxonomic group variation. While such a complex interplay may hinder our understanding of the plankton body size and trophic structure response to a rapidly changing world (Laughlin & Messier, 2015), analyses incorporating temperature, and focusing on different aspects of the community at once, as performed in this study, are crucial at community level.

### 4.3 The direct and indirect effects of ecological drivers on zooplankton body size and community trophic structure

Many studies have shown the importance of environmental factors for community trait variation (Brans et al., 2017; Sheridan & Bickford, 2011; Stark, Lehman, Crawford, Enquist, & Blonder, 2017), and most of these studies focused on fixed species trait means at a single time point (Bolnick et al., 2011). However, environmental conditions may vary over time and species may respond to this variation via genetic, plastic, or ontogenetic effects. Our temporal‐scale analysis is helpful to unraveling the direct and indirect effects of temperature on body size and trophic structure of zooplankton. Our study showed that body size of zooplankton community was influenced directly by temperature. This may reflect the fact that temperature can directly modify the food quality and fundamental physiological features of zooplankton taxa (Hart & Bychek, 2011). Trophic redundancy was the most important ecological driver linking environment and trophic structure in both mixing and stratification periods. Temperature influenced trophic structure directly, and this effect seemed to be more obvious at high temperature in the stratification period. The elevated water temperature had weakened the resilience of zooplankton community through altering trophic redundancy during water stratification period. The different responses of body size and trophic structure to altered environmental change between different periods make the ecosystem more complex and hard to predict. We anticipate to fill the knowledge gaps in illustrating the direct and indirect effects of warming on body size and trophic structure of zooplankton community. Further investigations should consider the importance of intrataxonomic variation and trophic interactions to better understand the links between warming, body size, and community trophic structure in freshwater ecosystems.

## 5 CONCLUSIONS

In this study, we investigated temporal variation in body size and community trophic structure of zooplankton, as well as their key ecological drivers in a subtropical reservoir across a 1‐year study. We found a change in zooplankton community trophic structure from the mixing to stratification periods to which intrataxonomic trait variation was an important contributor. However, the importance of intra‐ and intertaxonomic components greatly varied along the temperature change during the mixing and stratification periods. The different environmental conditions between the mixing and stratification periods resulted in heterogeneous responses of body size and trophic structure to the water temperature. Trophic redundancy was a key ecological factor linking environmental change and community trophic structure of zooplankton. However, different community properties (e.g., body size, community trophic redundancy, or trophic interaction) may show complex interactions and different responses to environmental change, and thus, it is important to consider the effect of various zooplankton properties (e.g., trophic redundancy) on community structure in future studies. We expect that the more frequent and severe extreme weather events predicted during the 21st century will exhibit prolonged and greater effects on community trait and trophic structure. Therefore, understanding how body size and community trophic structure respond to continued temperature fluctuation will be critical in guiding positive action to the future challenges in aquatic ecosystem management and conservation.

## CONFLICT OF INTEREST

None declared.

## AUTHOR CONTRIBUTIONS

J.Y. conceived the ideas and designed the experiments; X.G., H.C. and W.W. collected the samples; X.G. and J.Y. analyzed the data with input from L.G.; X.G. and J.Y. led the writing of the manuscript. All authors contributed critically to the drafts and gave final approval for publication.

## Data Availability

All data are included in the manuscript.
