# Supplementary material for: Responses of zooplankton body size and community trophic structure to temperature change in a subtropical reservoir
Source: Ecol Evol. 2019 Oct 29;9(22):12544–55. doi: 10.1002/ece3.5718 (PMC6875572; doi:10.1002/ece3.5718)
Supplement: Supplementary file 1 [file ECE3-9-12544-s001.pdf]

Journal: Ecology and Evolution

Supplementary information of the article:

Responses of zooplankton body size and community trophic structure to temperature change in a subtropical reservoir

Xiaofei Gao[1, 2, 3], Huihuang Chen[1, 2], Lynn Govaert[4, 5, 6], Wenping Wang[1, 2, 3], Jun Yang[1, 2,*]

[1] *Aquatic EcoHealth Group, Key Laboratory of Urban Environment and Health, Institute of Urban Environment, Chinese Academy of Sciences, Xiamen 361021, China*

[2] *Fujian Key Laboratory of Watershed Ecology, Institute of Urban Environment, Chinese Academy of Sciences, Xiamen 361021, China*

[3] *University of Chinese Academy of Sciences, Beijing 100049, China*

[4] *Department of Evolutionary Biology and Environmental Studies, University of Zurich, Winterthurerstrasse 190, CH-8057 Zürich, Switzerland*

[5] *Department of Aquatic Ecology, Eawag: Swiss Federal Institute of Aquatic Science and Technology, Überlandstrasse 133, 8600, Dübendorf, Switzerland*

[6] *Laboratory of Aquatic Ecology, Evolution and Conservation, KU Leuven, Charles Deberiotstraat, B-3000 Leuven, Belgium*

Running head: Temperature alters zooplankton trophic structure

Key words: community ecology; plankton; subtropical reservoir; temperature; trait variation; trophic structure

***Correspondence:** Jun Yang, Email: jyang@iue.ac.cn

This supplementary information contains:

- 15 Pages
- 13 Figures including one graphical abstract
- 1 Table

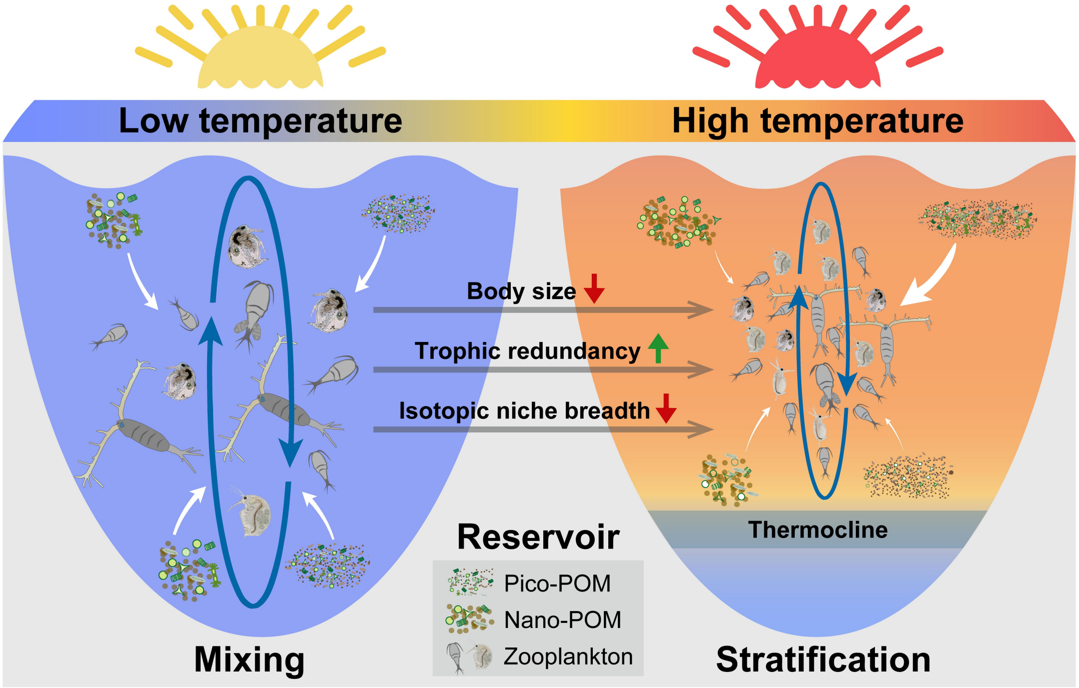

Graphical abstract

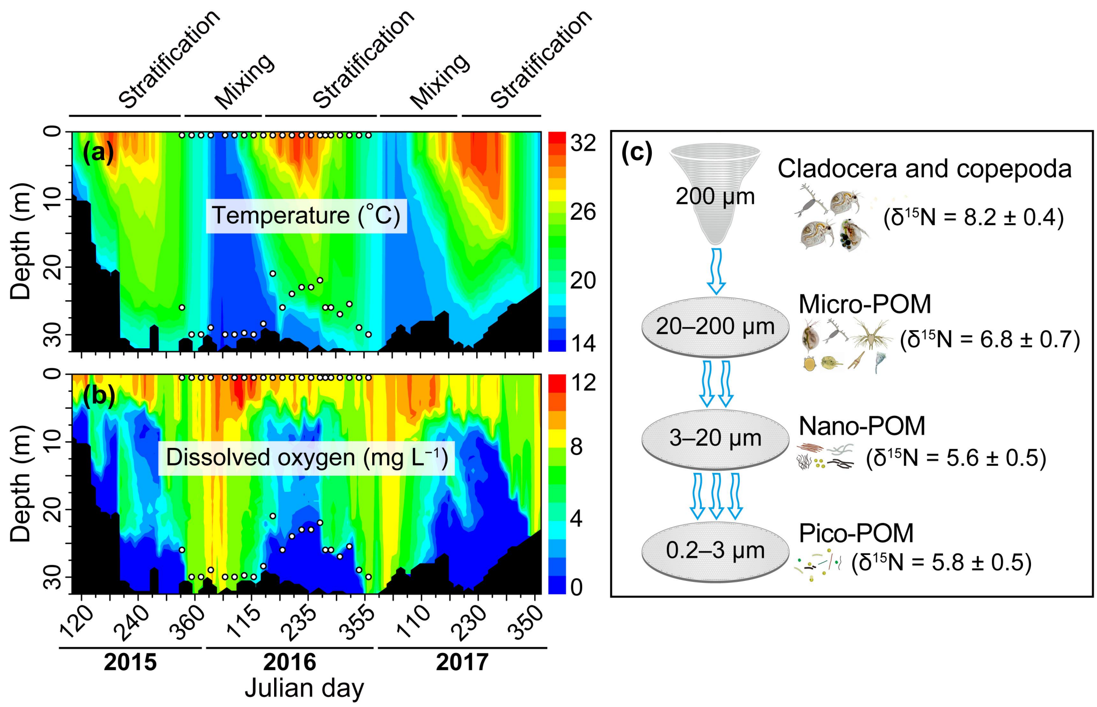

Fig. S1 Depth-time profiles of water temperature (**a**) and dissolved oxygen (**b**) in Tingxi Reservoir. White dots indicate the sampling depth and time. (**c**) $\delta^{15}N$ of zooplankton (cladocera and copepoda), micro-POM, nano-POM and pico-POM, respectively. Cladocerans and copepods were sampled by vertically hauling a plankton net from the bottom to the surface waters

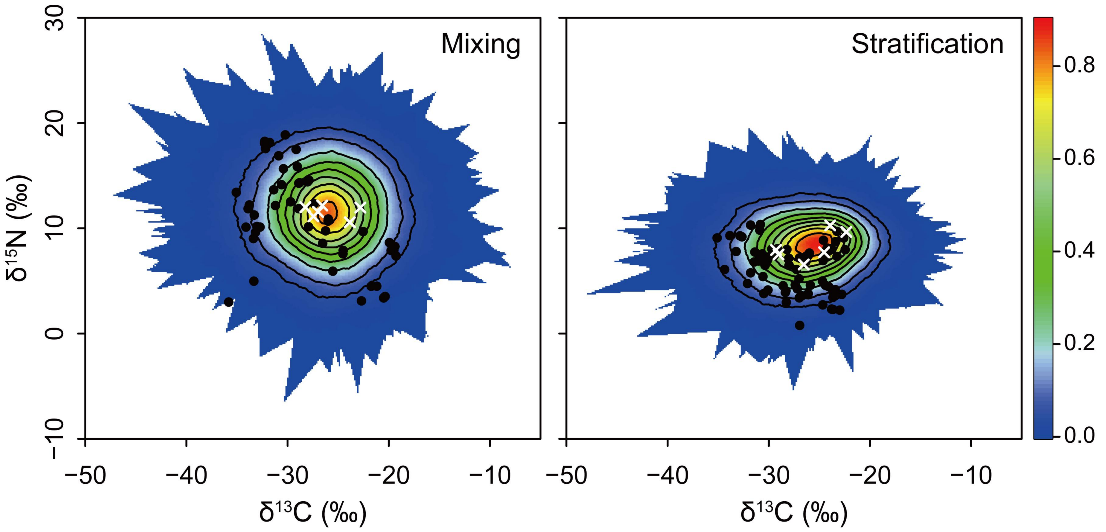

Fig. S2. The simulated mixing region of isotopic biplots for the water mixing and stratification periods in Tingxi Reservoir. The positions of the zooplankton taxa (black dots) and the average POMs signatures (white crosses) are shown. Probability contours are at the 5% level (outermost contour) and at every 10% level. Note that twelve and five zooplankton samples from mixing and stratification periods, respectively, were removed from the final mixing model as presented in the main text

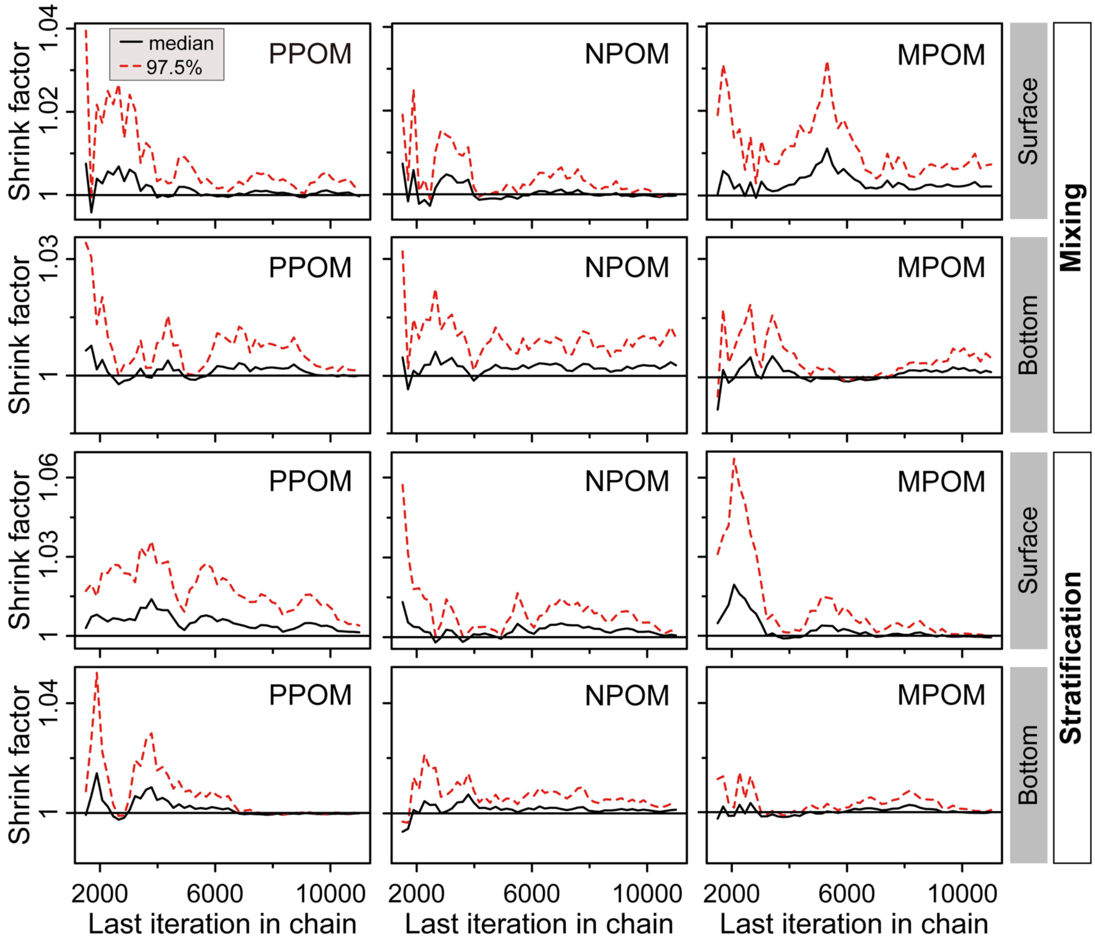

Fig. S3. Convergence diagnostics were created through Markov chain Monte Carlo (MCMC). The diagnostics can help to find those values which fit the data best and insure the model run properly. The shrink factor value close to 1 means that the mixing model has converged properly. If the value is above 1.1, it requires a longer iteration. The PPOM, NPOM and MPOM represent pico-, nano- and micro-POMs, respectively

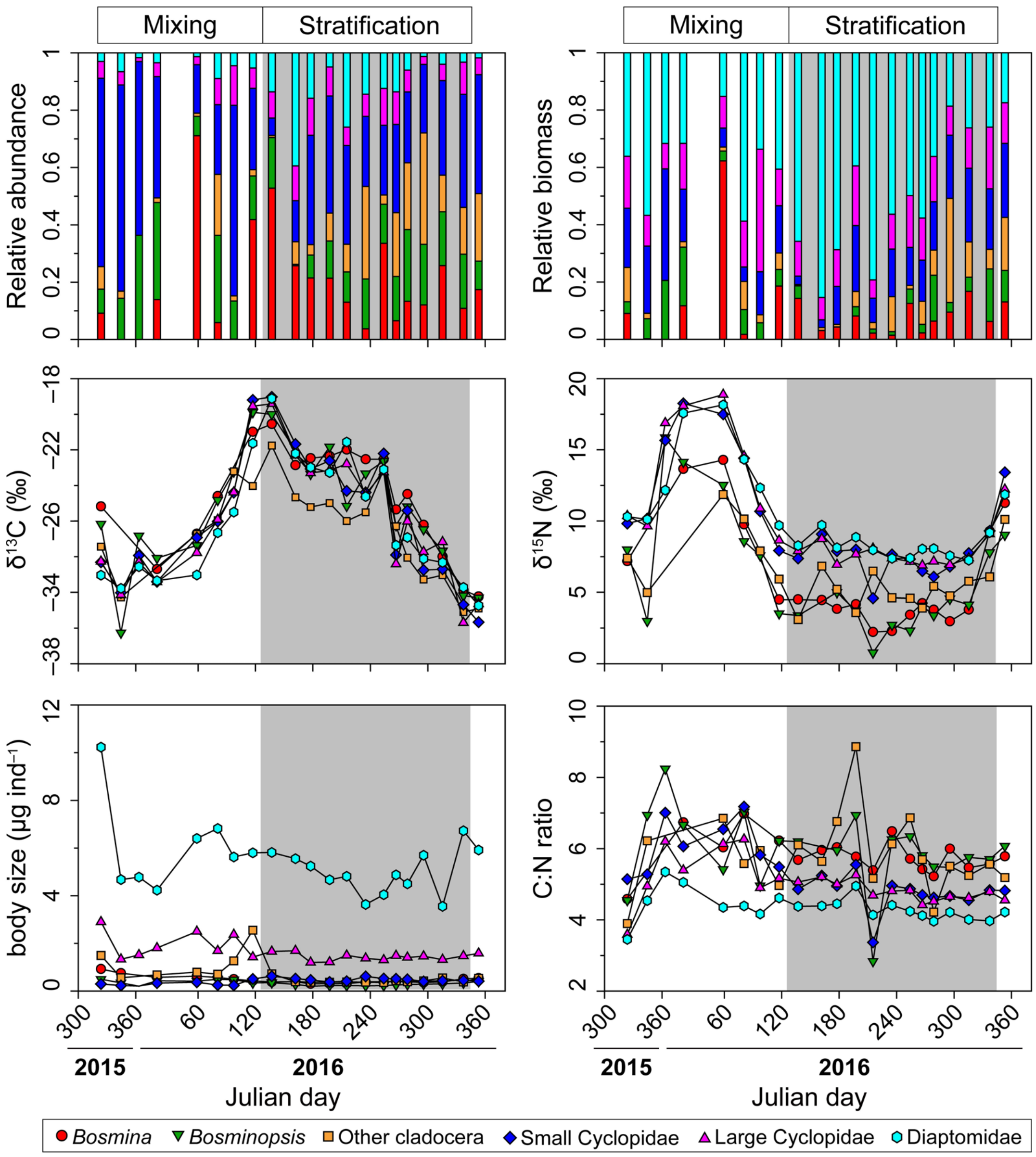

Fig. S4. Time series of relative abundance, relative biomass, $\delta^{13}C$, $\delta^{15}N$, body size (dry weight) and C:N ratio of different zooplankton taxonomic groups in Tingxi Reservoir. The shade zone represents thermal stratification period. The $\delta^{13}C$ increased during the mixing period, while it decreased during the stratification period for all zooplankton. On the contrary, $\delta^{15}N$ rapidly increased and decreased during the mixing period for all zooplankton, but stayed relatively similar during the stratification period. Diaptomidae were the largest zooplankton found in this study with the overall highest relative biomass (85%), but specifically peaked in its relative abundance (39%) at the beginning of the stratification period

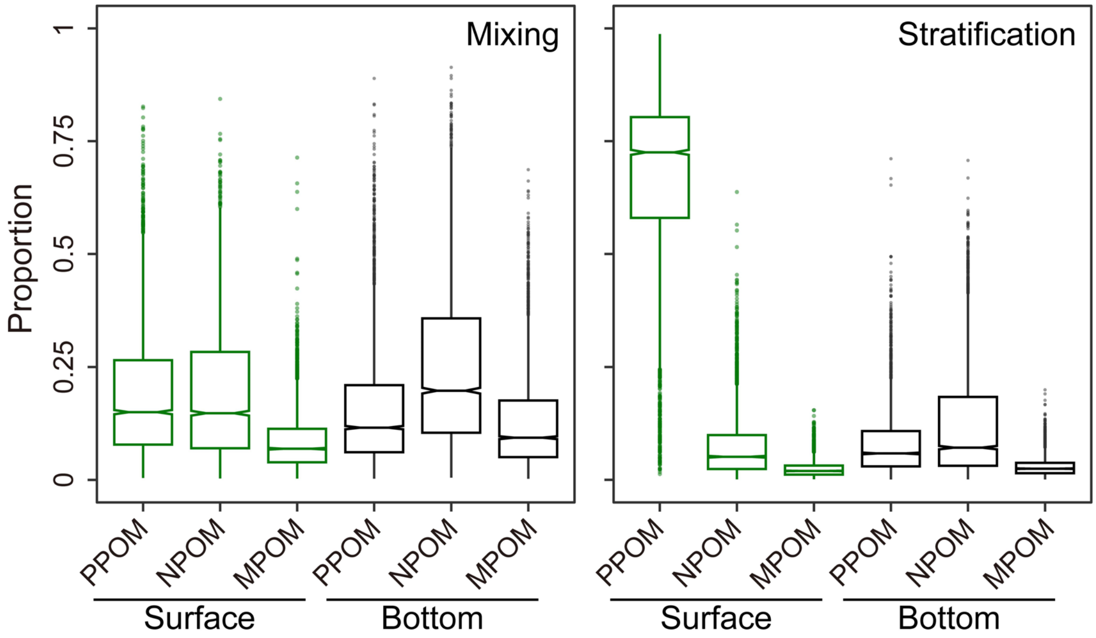

Fig. S5. Boxplots summarizing the contribution of pico-, nano- and micro-POMs to zooplankton community from water mixing and stratification periods in Tingxi Reservoir, respectively. Boxes and error bars represent the $25^{th}/75^{th}$ and $5^{th}/95^{th}$ percentiles, respectively. Middle line of the boxes represents the median of estimates. PPOM, NPOM and MPOM represent pico-, nano- and micro-POMs, respectively. Micro-POM showed a relatively small contribution to the diet of zooplankton community compared to pico-POM and nano-POM in both the mixing and stratification periods

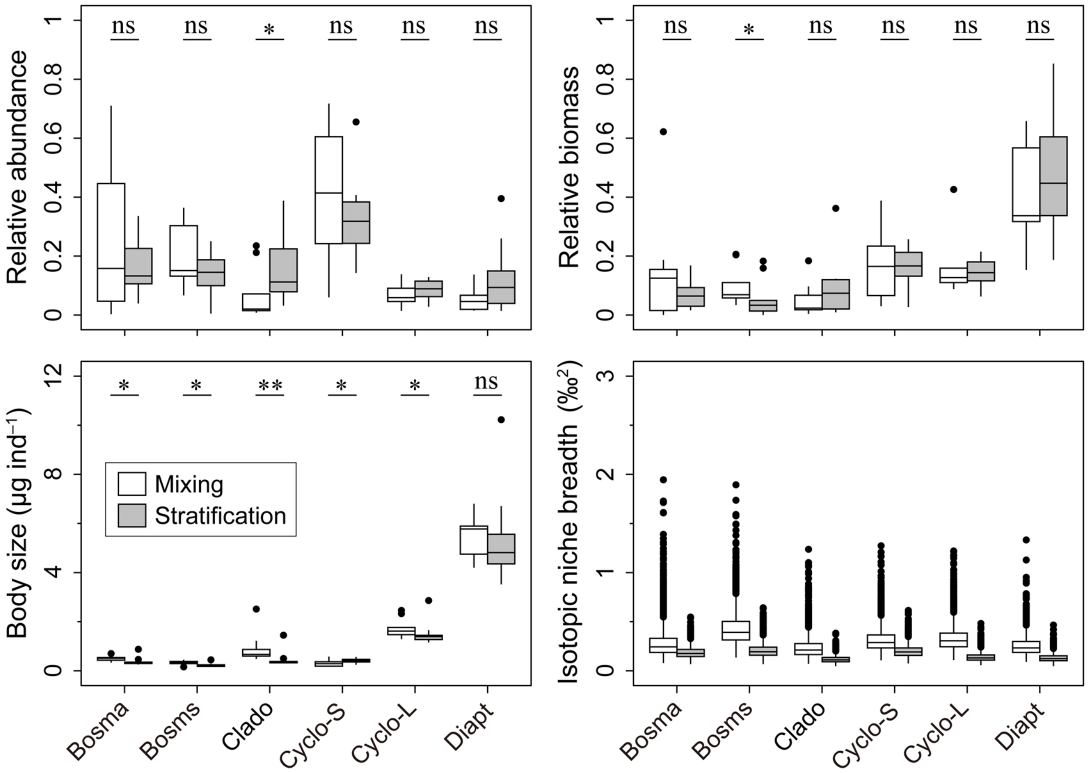

Fig. S6. Comparison of relative abundance, relative biomass, body size (dry weight) and variation in isotopic niche breadth (SEA_B) of different zooplankton taxonomic groups between water mixing (white box) and stratification (gray box) periods in Tingxi Reservoir. Bosma, *Bosmina*; Bosms, *Bosminopsis*; Clado, other Cladocera; Cyclo-S, small Cyclopidae (200–450 μm); Cyclo-L, large Cyclopidae (≥ 450 μm); Diapt, Diaptomidae. SEA_B represents the mode value of the Markov chain Monte Carlo (MCMC) output for each zooplankton taxonomic group. Statistical analysis is nonparametric Mann-Whitney *U* test, *$P < 0.05$, **$P < 0.01$

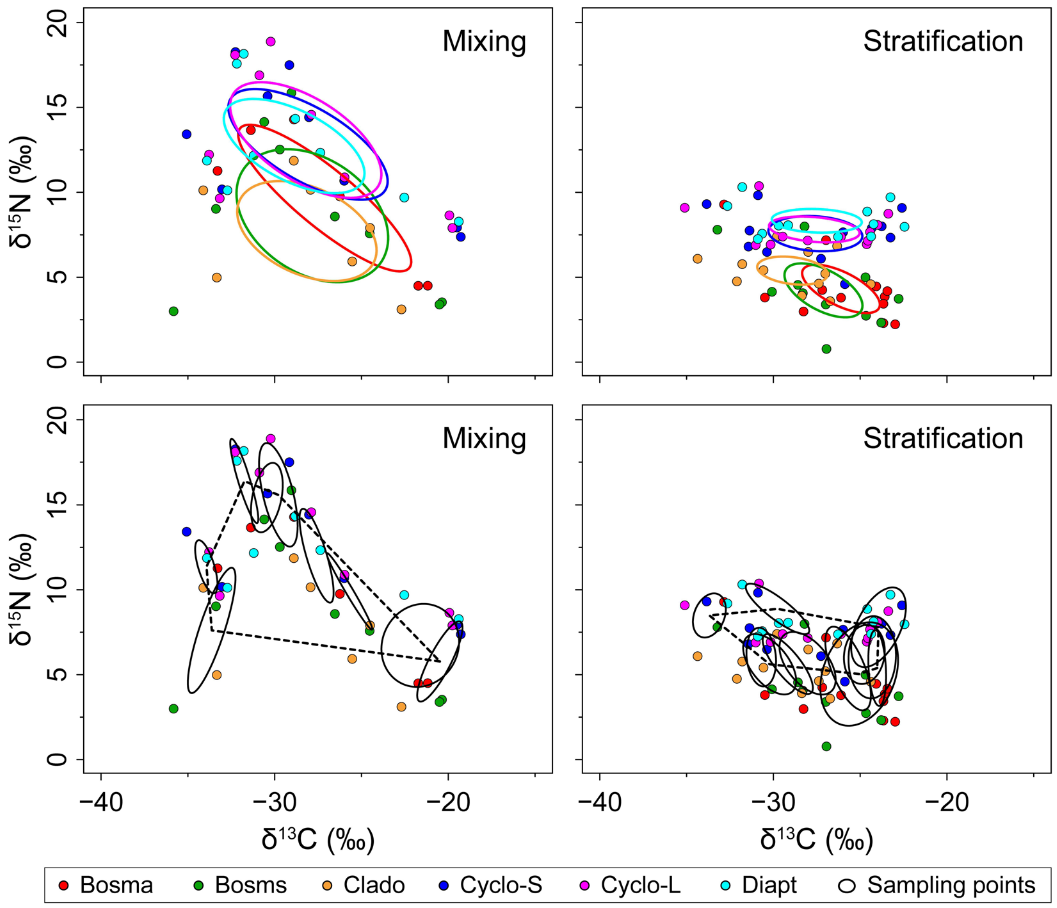

Fig. S7. Biplot of $\delta^{13}C$ and $\delta^{15}N$ values of zooplankton in water mixing and stratification periods (top panels), as well as the isotopic niche (SEA_C) distributions of different sampling time points (bottom panels). Ellipses in the top panel represent the isotopic niche breadth (SEA_C) of each zooplankton taxonomic group with 95% confidence interval of their bivariate mean. The black ellipses (bottom panels) represent SEA_C of different sampling time points. Zooplankton community significantly reduced its isotopic range going from the mixing period to stratification period. SEA_C is not corrected for source isotopic values and should only be used for comparison among zooplankton taxa within each period. Bosma, *Bosmina*; Bosms, *Bosminopsis*; Clado, other Cladocera; Cyclo-S, small Cyclopidae (200–450 μm); Cyclo-L, large Cyclopidae (≥ 450 μm); Diapt, Diaptomidae

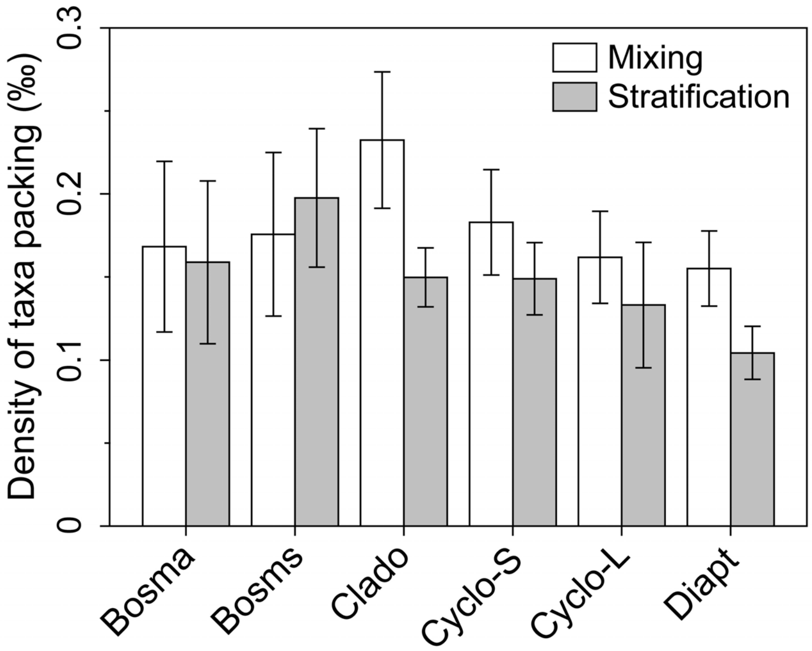

Fig. S8. Barplots showing the density of taxa packing (MNND ± SE) for each zooplankton taxonomic group during mixing (white) and stratification (gray) periods in Tingxi Reservoir. The mean nearest neighbour distance (MNND) was calculated for each zooplankton taxonomic group in the mixing and stratification periods as the mean of the Euclidean distance to each sample's nearest neighbour distance. Bosma, *Bosmina*; Bosms, *Bosminopsis*; Clado, other Cladocera; Cyclo-S, small Cyclopidae (200–450 µm); Cyclo-L, large Cyclopidae (≥ 450 µm); Diapt, Diaptomidae

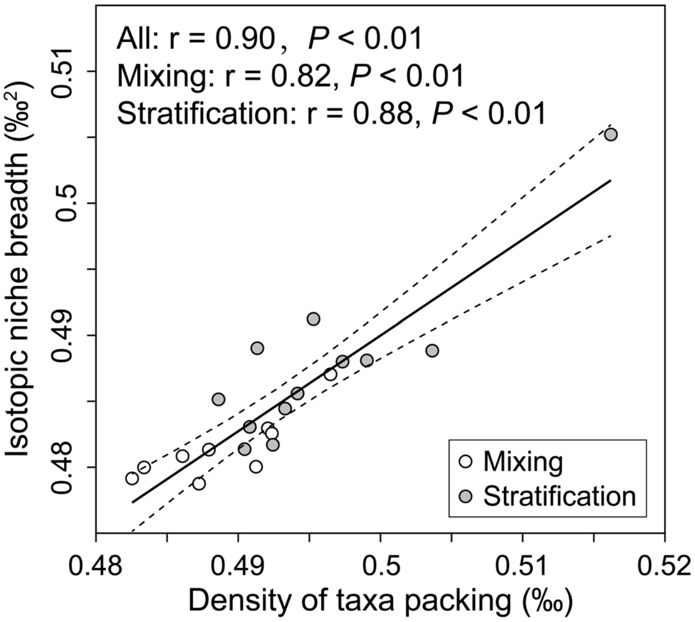

Fig. S9. The relationship between the density of taxa packing (MNND) and isotopic niches breadth (SEA_B) during the mixing and stratification periods in Tingxi Reservoir (n = 21). SEA_B represent the mode values of the MCMC output for each sample. The mean nearest neighbour distance (MNND) was calculated for each sample in the mixing and stratification periods. White and gray circles represent the water mixing and stratification periods, respectively. The continuous line represents a linear logistic regression curve with its 95% confidence interval (dashed line) for all sampling points

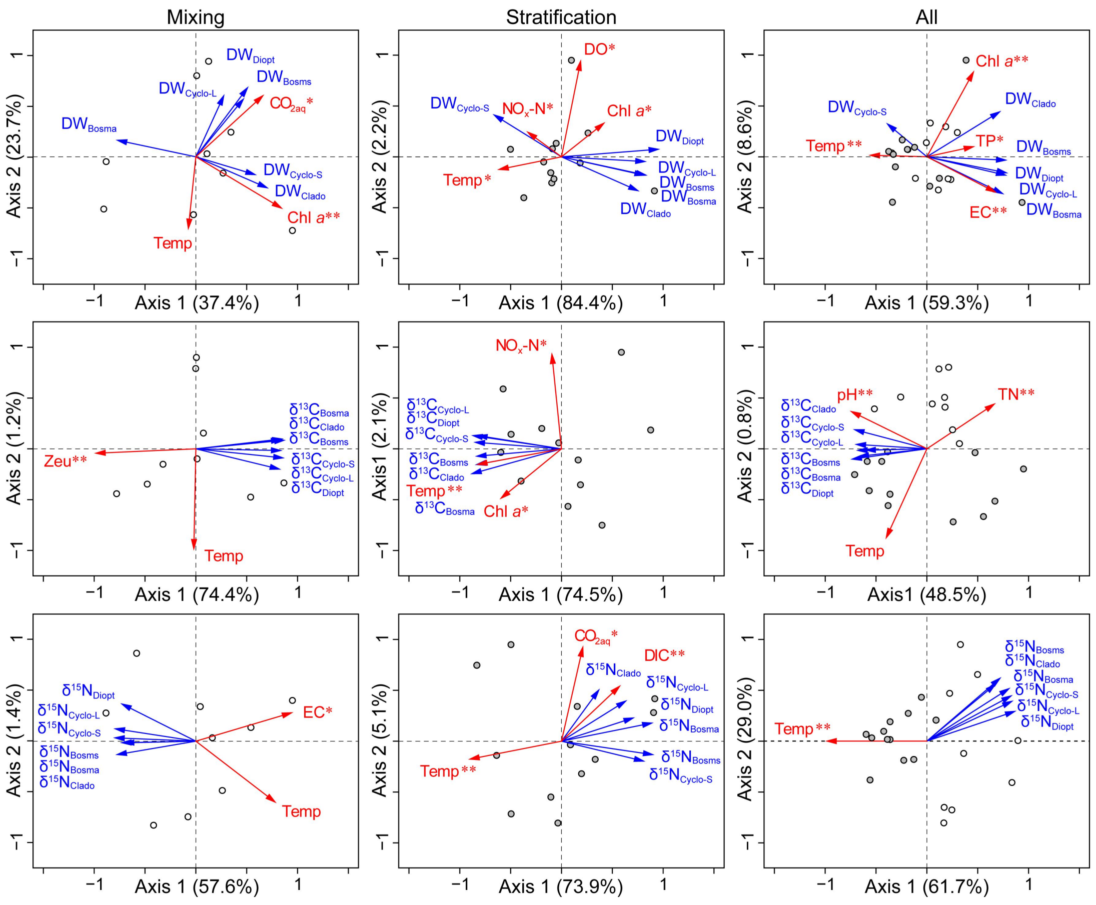

Fig. S10. RDA plots showing the relationships between significant local environmental factors and zooplankton body size (DW) or community trophic structure (represented by $\delta^{13}C$ and $\delta^{15}N$, respectively). All data were log-transformed except pH. Temp, water temperature; DO, dissolved oxygen; CO_{2aq}, dissolved CO_2 concentration of water; Chl *a*, chlorophyll *a*; EC, electrical conductivity; Zeu, euphotic depth; DIC, dissolved inorganic carbon; TN, total nitrogen; NO_x-N, nitrate and nitrite nitrogen; TP, total phosphorus. DW_{Bosma}, DW_{Bosms}, DW_{Clado}, $DW_{Cyclo\text{-}S}$, $DW_{Cyclo\text{-}L}$ and DW_{Diapt} represent body size (dry weight) of *Bosmina*, *Bosminopsis*, other Cladocera, small Cyclopidae (200–450 μm), large Cyclopidae (≥ 450 μm) and Diaptomidae, respectively. *$P < 0.05$, **$P < 0.01$

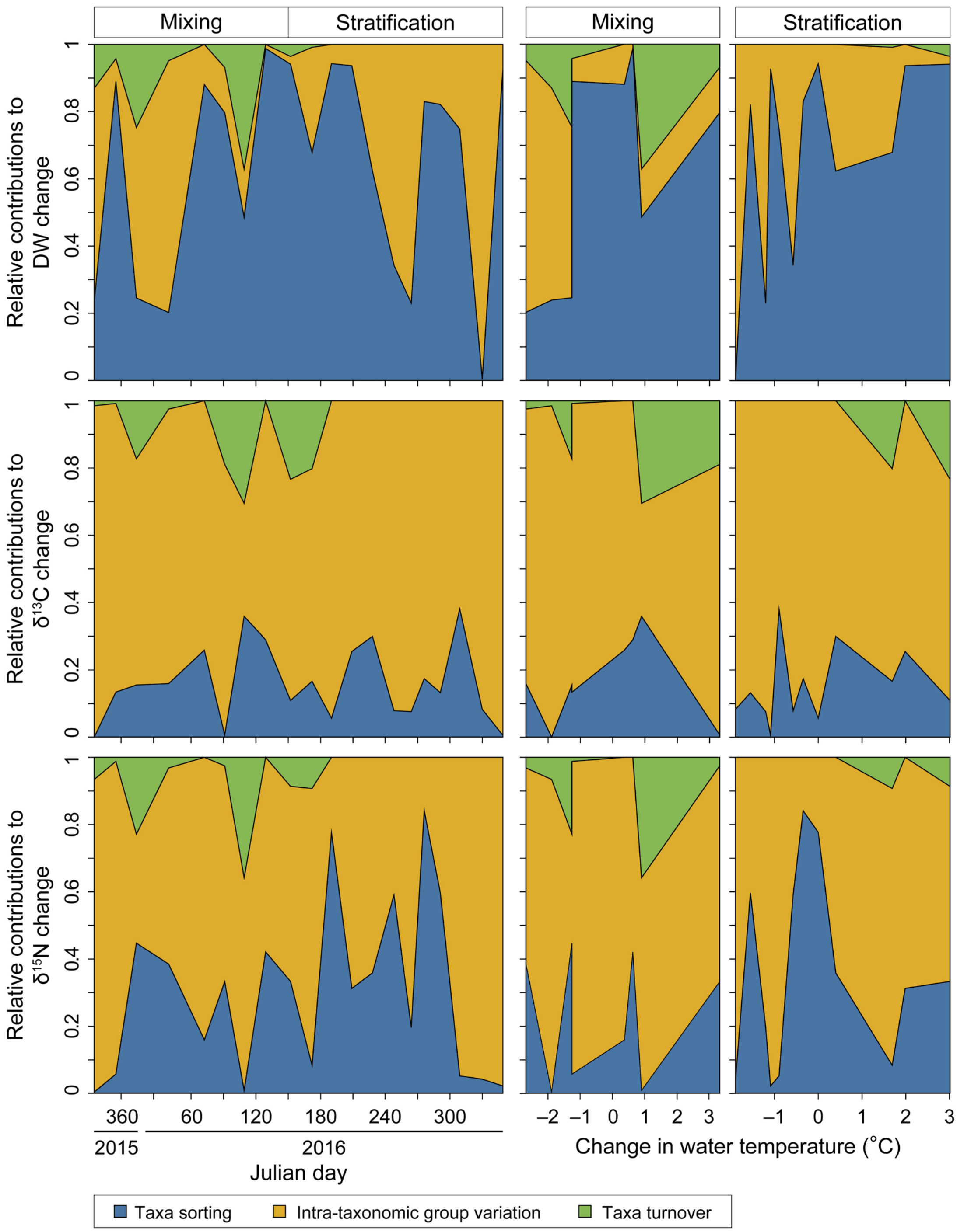

Fig. S11. We performed a partitioning to the observed temporal changes in zooplankton body size (DW), $\delta^{13}C$ and $\delta^{15}N$, respectively. First column represents the contribution of taxa sorting (blue), intra-taxonomic trait variation (orange) and taxa turnover (green) to changes in DW, $\delta^{13}C$ and $\delta^{15}N$ across the sampling period, respectively. Second and third columns show the same contributions but now plotted against change in water temperature between consecutive sampling points during the mixing and stratification periods, respectively. Here, the changes in $\delta^{13}C$ and $\delta^{15}N$ are the index of trophic structure variation of each taxonomic group

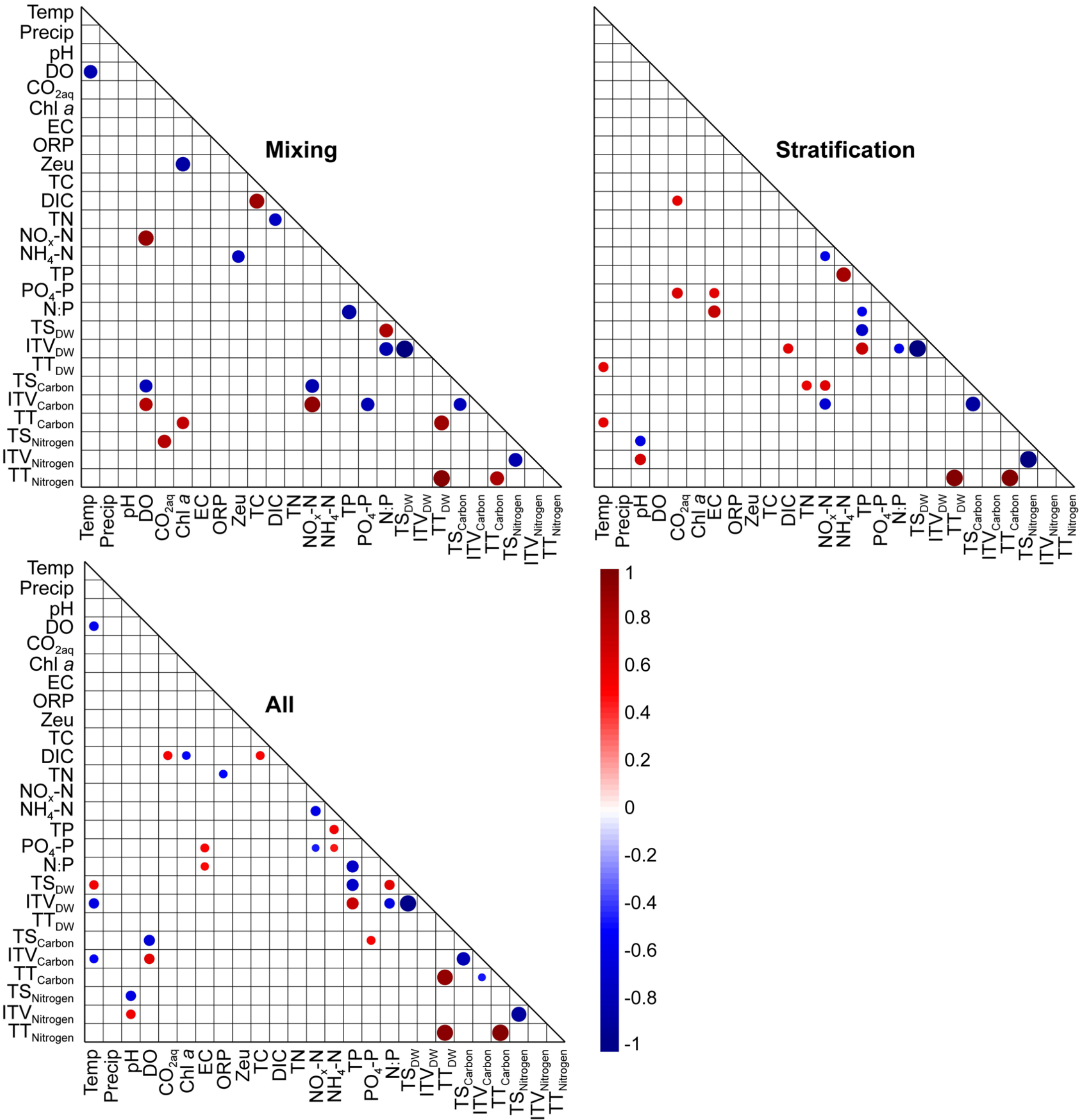

Fig. S12. Spearman's correlation coefficients for change in environmental data and the relative contributions of different components for the change in body size (DW), $\delta^{13}C$ and $\delta^{15}N$ of zooplankton in Tingxi Reservoir. Temp, water temperature; Precip, total precipitation for twenty days before sampling; DO, dissolved oxygen; CO_{2aq}, dissolved CO_2 concentration of water; Chl *a*, chlorophyll *a*; EC, electrical conductivity; ORP, oxidation-reduction potential; Zeu, euphotic depth; TC, total carbon; DIC, dissolved inorganic carbon; TN, total nitrogen; NO_x-N, nitrate and nitrite nitrogen; NH_4-N, ammonium nitrogen; TP, total phosphorus; PO_4-P, phosphate phosphorus; N:P, TN:TP ratio; TS_{DW}, ITV_{DW}, TT_{DW} represent contributions of taxa sorting, intra-taxonomic group variation, and taxa turnover to body size change, respectively. TS_{Carbon}, ITV_{Carbon}, TT_{Carbon} represent contributions of taxa sorting, intra-taxonomic group variation, and taxa turnover to $\delta^{13}C$ change, respectively. $TS_{Nitrogen}$, $ITV_{Nitrogen}$, $TT_{Nitrogen}$ represent contributions of taxa sorting, intra-taxonomic group variation, and taxa turnover to $\delta^{15}N$ change of zooplankton, respectively. We only presented the significant correlations ($P < 0.05$) for simplicity

Table S1 The main species or genera of zooplankton in Tingxi Reservoir

Taxonomic group	Species
Bosmina	*Bosmina longirostris*
Bosminopsis	*Bosminopsis deitersi*
Other cladocera	*Ceriodaphnia cornuta*
	Ceriodaphnia quadrangula
	Chydorus sphaericus
	Diaphanosoma brachyurum
	Diaphanosoma leuchtenbergianum
	Moina macrocopa
	Moina micrura
Small cyclopidae (200–450 μm)	*Microcyclopa* sp.
Large cyclopidae (≥ 450 μm)	*Cyclops vicinus*
	Mesocyclops thermocyclopoides
	Thermocyclops taihokuensis
	Tropocyclops parvus
Diaptomidae	*Neutrodiaptomus tumidus*
	Phyllodiaptomus tunguidus
